# Treatment modification after starting cART in people living with HIV: retrospective analysis of the German ClinSurv HIV Cohort 2005–2017

**DOI:** 10.1007/s15010-020-01469-6

**Published:** 2020-07-01

**Authors:** Melanie Stecher, Philipp Schommers, Christian Kollan, Matthias Stoll, Frieder Kuhlendahl, Hans-Jürgen Stellbrink, Jan-Christian Wasmuth, Christoph Stephan, Laura Hamacher, Clara Lehmann, Christoph Boesecke, Johannes Bogner, Stefan Esser, Carlos Fritzsche, Annette Haberl, Dirk Schürmann, Olaf Degen, Heinz-August Horst, Christian Hoffmann, Björn Jensen, Carolynne Schwarze-Zander, Martin Platten, Gerd Fätkenheuer, Daniel Schmidt, Barbara Gunsenheimer-Bartmeyer, Jörg Janne Vehreschild, On behalf of the ClinSurv Study Group

**Affiliations:** 1 Department I for Internal Medicine, Faculty of Medicine and University Hospital Cologne, University of Cologne, Herderstraße 52-54, 50931 Cologne, Germany; 2 German Center for Infection Research (DZIF), Partner Site Bonn-Cologne, Cologne, Germany; 3 Robert-Koch Institute (RKI), Berlin, Germany; 4 Department of Clinical Immunology and Rheumatology, Hannover Medical School, Hannover, Germany; 5 Ifi-Institute for Interdisciplinary Medicine, Hamburg, Germany; 6 Infectious Disease Medical Center, Hamburg, Germany; 7 Department I for Internal Medicine, University Hospital of Bonn, Bonn, Germany; 8 German Center for Infection Research (DZIF), Partner Site Bonn-Cologne, Bonn, Germany; 9 Department of Infectious Diseases, University Hospital Frankfurt, Goethe-University, Frankfurt am Main, Germany; 10 Section for Infectious Diseases, Medical Clinic and Polyclinic IV, University Hospital, Ludwig-Maximilians-University Munich, Munich, Germany; 11 Clinic of Dermatology, Department of Venerology, University Hospital Essen, Essen, Germany; 12 Department of Tropical Medicine and Infectious Diseases, University of Rostock, Rostock, Germany; 13 ICH Study Center, Infektionsmedizinisches Centrum Hamburg, Hamburg, Germany; 14 Department of Gastroenterology, Hepatology and Infectiology, University of Düsseldorf, Düsseldorf, Germany; 15 Labor Dr. Wisplinghoff, Cologne, Germany; 16 Charité - University Medicine Berlin, Berlin, Germany; 17 Department of Hematology and Oncology, Johann Wolfgang Goethe University of Frankfurt, Frankfurt am Main, Germany; 18 University Clinic Hamburg Eppendorf, Hamburg, Germany; 19 University Hospital Schleswig–Holstein, Kiel, Germany

**Keywords:** HIV, cART, Treatment modification, First-line regimen

## Abstract

**Objective:**

Combination antiretroviral therapy (cART) has markedly increased survival and quality of life in people living with HIV. With the advent of new treatment options, including single-tablet regimens, durability and efficacy of first-line cART regimens are evolving.

**Methods:**

We analyzed data from the prospective multicenter German Clinical Surveillance of HIV Disease (ClinSurv) cohort of the Robert-Koch Institute. Kaplan–Meier and Cox proportional hazards models were run to examine the factors associated with treatment modification. Recovery after treatment initiation was analyzed comparing pre-cART viral load and CD4+ T-cell counts with follow-up data.

**Results:**

We included 8788 patients who initiated cART between 2005 and 2017. The sample population was predominantly male (*n* = 7040; 80.1%), of whom 4470 (63.5%) were reporting sex with men as the transmission risk factor. Overall, 4210 (47.9%) patients modified their first-line cART after a median time of 63 months (IQR 59–66). Regimens containing integrase strand transfer inhibitors (INSTI) were associated with significantly lower rates of treatment modification (adjusted hazard ratio 0.44; 95% CI 0.39–0.50) compared to protease inhibitor (PI)-based regimens. We found a decreased durability of first-line cART significantly associated with being female, a low CD4+ T-cell count, cART initiation in the later period (2011–2017), being on a multi-tablet regimen (MTR).

**Conclusions:**

Drug class and MTRs are significantly associated with treatment modification. INSTI-based regimens showed to be superior compared to PI-based regimens in terms of durability.

**Electronic supplementary material:**

The online version of this article (10.1007/s15010-020-01469-6) contains supplementary material, which is available to authorized users.

## Introduction

Combination antiretroviral therapy (cART) has improved markedly over the past decades. Today, people living with HIV (PLWH) can mostly be treated with safe and well-tolerated cART leading to a long-term suppression of viremia [1], which results in a significant reduction of morbidity and mortality in PLWH. New drug combinations are available as once-daily or single-tablet regimens (STR) that improve adherence to cART and consequently lead to successful suppression of viremia [2]. An effective virological control and immunological reconstitution is crucial for therapeutic long-term management in PLWH. Reports from the United States of America (USA) of the early cART era found a median duration of the first-line cART regimen between 1 and 3 years, depending on the observed periods ranging from 1996 to 2009 [3–6]. A recent study found that this time was extended to almost 5 years in the period between 2008 and 2011 [7]. Data from Australia showed a trend to fewer treatment modifications in recent years and demonstrated a stable rate of first-line treatment modifications, comparable to other cohorts [8]. Various studies found factors that are believed to lead to an earlier modification of the initial cART, including treatment with a protease inhibitor (PI), a high baseline HIV RNA level, and multiple-tablet regimens (MTR) as well as not receiving a once-daily cART or STR [3, 9, 10].

While several of these factors are conclusive and have been proven by different cohort studies, most of these studies have been done before the introduction of integrase strand transfer inhibitors (INSTI). The first INSTI, raltegravir (RAL), was approved by the Food and Drug Administration (FDA) in 2007 [11]. It was followed by elvitegravir (EVG), dolutegravir (DTG) [12], and bictegravir (BIC) [13], which were approved by the FDA in 2012, 2013, and 2018, respectively, and were subsequently approved in Germany [14]. In 2018, INSTI-based regimens for first-line treatment were recommended, among others, by the International Antiviral Society (IAS)-USA, the European AIDS Clinical Society (EACS), and German Austrian AIDS Society [1, 15, 16]. However, there are limited data on reasons to modify first-line regimens in the era of novel cART regimens in routine clinical care conditions.

Therefore, we examined the durability of different first-line cART regimens and characteristics of those who modified first-line cART in a real-world setting. We also aimed to describe the characteristics of those who achieved viral suppression after cART initiation, as found in the national German Clinical Surveillance of HIV Disease Cohort (ClinSurv) in Germany between 2005 and 2017.

## Methods

### Study design

This study was planned and conducted by the academic and public sector researchers of the University Hospital of Cologne and German Center for Infection Research. We conducted a retrospective analysis of PLWH enrolled in the German national ClinSurv HIV cohort of the Robert Koch Institute (RKI), an ongoing, long-term observational multicenter cohort initiated in 1999. Details of the ClinSurv HIV cohort have been published previously [17]. In brief, 15 German University Hospitals and specialized clinical HIV treatment centers contribute comprehensive data on treatment and outcomes to the ClinSurv cohort. Data for ClinSurv are collected upon enrollment and are updated biannually based on all visits to the participating centers.

### Study population

PLWH enrolled in ClinSurv from January 2005 through June 2017 were eligible for our analysis if they initiated their first-line therapy during this time period, were older than 18 years, and had both, an available pre-cART CD4+ T-cell count and viral load, yielding 8788 participants. Patients who received their first-line treatment in the setting of a clinical study were excluded from this analysis.

### Definition of the endpoint

The first endpoint was defined as the date when the first-line drug class was changed to another class during follow-up. Modification was not counted if individuals alternated the dosage or used other drugs within the same drug class or if they interrupted the first-line drug class. The second outcome was defined as immunological recovery after 12 months of first-line treatment. Patients were censored if they died during the study period, failed to follow-up, or reached the end of the observation period before experiencing the endpoint. The epidemiological trends were evaluated between 2005 and 2017 (date of starting the respective cART). Moreover, we compared an early period (2005–2010) and late period (2011–2017), with the latter representing the period in which current INSTI regimens and STRs become available.

### Statistical analyses

Baseline characteristics of included patients were reported as absolute numbers with percentage and median with interquartile range (IQR), as appropriate. Patients were compared during the period 2005–2010 (early period) and 2011–2017 (late period), using the Chi-square test.

We analyzed two different outcomes. First, we examined the durability of the first-line regimen, defined as switching the first-line cART drug class. Second, we analyzed viral suppression after cART initiation, defined as achieving low or undetectable plasma HIV RNA (< 200 copies/mL) after 12 months (± 6 months). The Kaplan–Meier (KM) method was used to examine factors independently associated with the durability of first-line cART. Differences between subgroups were compared by the log-rank test. The presence of multicollinearity problems was assessed among the explanatory variables using the Tolerance and Variance Inflating Factor [18]. A multivariable Cox regression model was used to identify the factors associated with the durability of the first-line cART regimen. We used backward selection eliminating variables with *p* > 0.2 to reach the simplest model that explained the data. Our final multivariable model was adjusted for sex, transmission risk group, pre-cART CD4+ T-cell count and viral load, first-line drug class, antiretroviral drugs that were included in the first-line regimen (TDF/FTC in combination with EFV, DRV/r, LPV/r, ATV/r, RAL, NVP, RPV, DTF or Others), the tablet regimen (single versus multi tablet regimen), and the period of cART initiation (2005–2010 and 2011–2017). Hazard ratio (HR) and adjusted hazard ratio (aHR) with 95% confidence intervals (CI) were reported to measure the strength and association between variables.

We further analyzed immunological recovery after first-line initiation. Pre-cART viral load and CD4+ T-cell count were compared to the closest measurement of month 12 (± 6 months). *P* values of < 0.05 were considered statistically significant. All analyses were compiled using STATA (Stata Statistical Software: Release 14. College Station, StataCorp LP, TX, USA).

### Ethical consideration

The RKI is the national public health institute and is responsible for disease prevention and control in Germany. The Federal Commissioner for Data Protection is the responsible entity for studies conducted by the RKI. All HIV infections are reported to the RKI as a statutory duty for anonymous notification, implemented by the national Protection against Infection Act. The data collected in the ClinSurv cohort are generated during routine care. In this scenario, no informed consent or permission for secondary analysis of the anonymized data was required. This study was performed in accordance with the Declaration of Helsinki.

## Results

### Study population

Between January 2005 and June 2017, a total of 8788 PLWH met the inclusion criteria and were eligible for our analyses (Figure S1). Baseline characteristics are presented in Table 1. The median age was 38 years (IQR 31–46), and patients were predominantly male (*n* = 7040; 80.1%). Among male patients, 4470 (63.5%) reported sex with men (MSM) as the main transmission risk factor. In the total study population, the median pretreatment CD4+ T-cell count was 241 cells/µL (IQR 111–369 cells/µL), and 39.6% of patients had a CD4+ T-cell count below 200 cells/µL. The median pretreatment HIV RNA was 65,000 copies/mL (IQR 13,903–213,000), and 38.9% of patients had a pretreatment viral load greater than 100,000 copies/mL. A total of 338 (3.8%) patients died over the observation period, of whom 177 (52.4%) were on a PI-based regimen, 105 (31.1%) on a nucleotide reverse-transcriptase inhibitor (NRTI)/non-nucleotide reverse-transcriptase inhibitor (NNRTI) regimen, and 22 (10.1%) on an INSTI-based regimen.

**Table 1:** Overall patient characteristics and comparing characteristics during the early (2005–2010) and late period (2011–2017)

	Patient characteristics, *n* (%)	2005–2010, *n* (%)	2011–2017, *n* (%)	*p* value*
Total	8788 (100)	4550 (51.8)	4238 (48.2)	
Age (median, IQR)	38 (31–46)	38 (31–46)	38 (30–47)	**0.025**
18–39	4629 (52.7)	2436 (55.5)	2193 (53.6)	
40–69	3770 (42.9)	1921 (43.8)	1849 (45.2)	
≥ 70	77 (0.9)	30 (0.7)	47 (1.1)	
Sex				0.109
Female	1748 (19.9)	935 (20.5)	813 (19.2)	
Male	7040 (80.1)	3615 (79.5	3425 (80.8)	
Region of origin				**<** **0.001**
Germany	6046 (68.8)	3218 (70.7)	2828 (66.7)	
Europe	993 (11.3)	441 (9.7)	552 (13.0)	
Middle East	90 (1.0)	39 (0.9)	51 (1.2)	
Sub-Saharan Africa	1005 (11.4)	533 (11.7)	472 (11.1)	
Asia, Australia and New-Zealand	254 (2.9)	152 (3.3)	102 (2.4)	
North and Latin America	222 (2.5)	118 (2.6)	104 (2.5)	
Others/unknown	178 (2.0)	49 (1.1)	129 (3.0)	
Risk group				**<** **0.001**
MSM	4470 (50.9)	2273 (50.0)	2197 (51.8)	
HTS	1467 (16.7)	279 (6.1)	172 (4.1)	
ENDEMIC	1171 (13.3)	748 (16.4)	719 (17.0)	
PWID	451 (5.1)	646 (16.4)	525 (12.4)	
Other/unknown	1229 (14.0)	604 (13.3)	625 (14.7)	
Pre-cART CD4+ T-cell count (µL)				**<** **0.001**
(Median, IQR)	241 (111–369)	215 (102–320)	280 (124–425)	
< 200	3479 (39.6)	2014 (45.9)	1465 (35.6)	
200–349	2631 (29.9)	1503 (34.3)	1128 (27.4)	
350–499	1399 (15.9)	575 (13.1)	824 (20.0)	
≥ 500	992 (11.3)	295 (6.7)	697 (16.9)	
Pre-cART HIV-1 RNA viral load (copies/mL) (median, IQR)	65,000 (13,903–213,000)	68,575 (15,276–223,904)	60,400 (12,500–205,317)	0.114
< 200	196 (2.2)	104 (2.5)	924 (2.3)	
201–5000	939 (10.7)	494 (11.7)	445 (11.3)	
5001–100,000	3638 (41.4)	1838 (43.6)	1800 (45.7)	
100,001–1 Mio	2911 (33.1)	1550 (36.8)	1361 (34.5)	
> 1 Mio	474 (5.4)	229 (5.4)	245 (6.2)	
Durability of first-line in months (IQR)	63 (59–66)	68 (64–72)	52 (48–55)	**<** **0.001**
First-line drug class				**<** **0.001**
NRTI/PI/boosted	3682 (41.9)	2140 (47.0)	1542 (36.4)	
NRTI/NNRTI	2951 (33.6)	1945 (42.7)	1006 (23.7)	
NRTI/INSTI	1676 (19.1)	146 (3.2)	1530 (36.1)	
Others	479 (5.5) (4.4)	319 (7.0)	160 (3.8)	
Substance of the first-line regimen				**<** **0.001**
TDF/FTC/EFV	1734 (19.7)	1285 (28.2)	449 (10.6)	
TDF/FTC/DRV/r	1180 (13.4)	320 (7.0)	860 (20.3)	
TDF/FTC/LPV/r	863 (9.8)	708 (15.6)	155 (3.7)	
TDF/FTC/ATV/r	655 (7.5)	301 (6.6)	354 (8.4)	
TDF/FTC/RAL	515 (5.9)	116 (2.5)	399 (9.4)	
TDF/FTC/NVP	469 (5.3)	348 (7.6)	121 (2.9)	
TDF/FTC/RPV	367 (4.2)	18 (0.4)	349 (8.2)	
TDF/FTC/DTG	352 (4.0)	15 (0.3)	337 (8.0)	
Others	2653 (30.2)	1439 (31.6)	1214 (28.6)	
Number of tablets per day				**<** **0.001**
1	1113 (13)	93 (2.1)	1020 (24.5)	
2–3	4614 (53.7)	2283 (51.6)	2331 (55.9)	
4–9	2796 (32.5)	1983 (44.8)	813 (19.5)	
≥ 10	69 (0.8)	66 (1.5)	3 (0.1)	
Single tablet regimen				0.272
STR	2472 (28.1)	1303 (28.6)	1169 (27.6)	
MTR	6316 (71.9)	3247 (71.4)	3069 (72.4)	
Regimen with INSTIs				**<** **0.001**
RAL	653 (7.4)	144 (88.9)	509 (31.9)	
DTG	746 (8.5)	17 (10.5)	729 (45.7)	
EVG	359 (4.1)	1 (0.6)	358 (22.4)	
Tablet intake				**<** **0.001**
Once per day	6063 (69.0)	2547 (41.3)	3516 (83.0)	
Twice per day	2529 (28.8)	1878 (41.3)	651 (15.4)	
Reason for discontinuing first-line therapy				**<** **0.001**
Side effects of drugs	792 (9.0)	514 (11.3)	278 (6.6)	
Simplification of therapy	394 (4.5)	192 (4.2)	202 (4.8)	
Patients’ choice	267 (3.0)	168 (3.7)	99 (2.3)	
Decision of the responsible physician	259 (2.9)	128 (2.8)	131 (3.1)	
Non-adherence	212 (2.4)	120 (2.6)	92 (2.2)	
Concomitant diseases	136 (1.5)	80 (1.8)	56 (1.3)	
Virological failure	133 (1.5)	85 (1.9)	48 (1.1)	
Others	462 (5.3)	276 (6.1)	186 (4.4)	

*p* values of < 0.05 in bold depict significant results

Risk group: *MSM* men who have sex with men, *HTS* heterosexual, *ENDEMIC* recent immigration from a country with a high HIV prevalence > 1%, *PWID* people who inject drugs. First-line drug class: *NRTI* nucleoside reverse- transcriptase inhibitor, *NNRTI* non-nucleoside reverse-transcriptase inhibitors, *INSTI* integrase strand transfer inhibitors, *PI* protease inhibitor. Substance of first-line regimen: *TDF* tenofovir, *FTC* emtricitabine, *EFV* efavirenz, *DRV* darunavir, *ATV* atazanavir, *RAL* raltegravir, *NVP* nevirapine, *RPV* rilpivirine, *DTG* dolutegravir. Regime: *STR* single-tablet regimen, *MTR* multi-tablet regimen. First-line with INSTI regimen: *RAL* raltegravir, *EVG* elvitegravir, *DLG* dolutegravir

*Chi-Square test (*p* < 0.05)

**Fig. 1:**
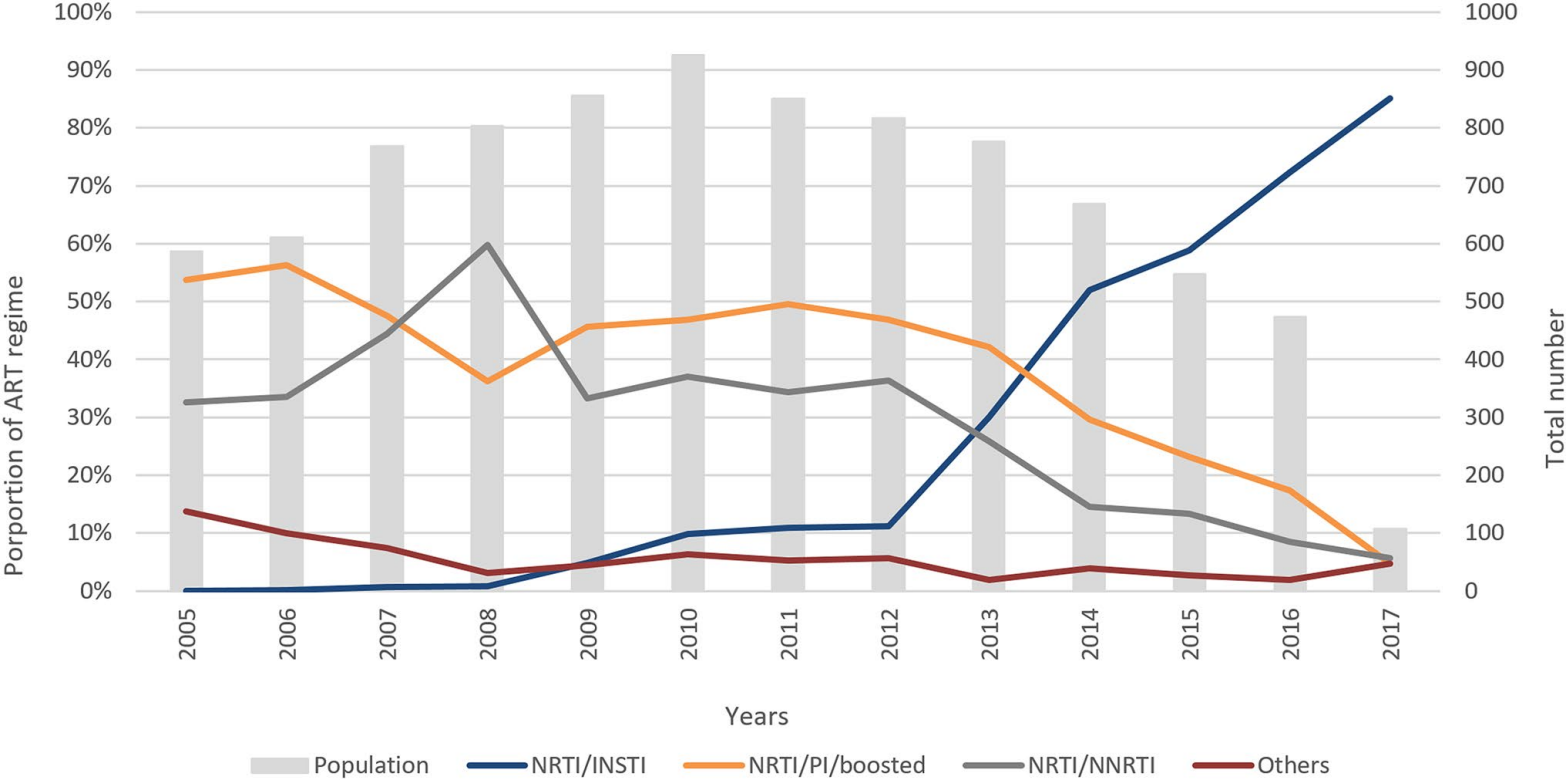
First-line cART regimen and total number of patients over time. Proportions of each cART regimen (vertical axis on the left side) are shown in three lines. NRTI/INSTI (nucleoside reverse-transcriptase inhibitor/integrase strand transfer inhibitor) (blue), NRTI/PI/boosted (non-nucleoside reverse-transcriptase inhibitors/protease inhibitor/boosted) (orange), NRTI/NNRTI (nucleoside reverse-transcriptase inhibitor/non-nucleoside reverse-transcriptase inhibitors) (gray), and others (red). The gray bars representing the total number of patients starting cART in the respective year (vertical axis on the right side)

The most common prescribed first-line cART regimens were TDF/FTC/EFV (*n* = 1734/8788; 19.7%) and TDF/FTC/DRV/r (*n* = 1180/8788; 13.4%). Within the early period of 2005–2010, TDF/FTC/EFV accounted for almost one third (*n* = 1285/4450; 28.2%) of all prescribed cARTs, while TDF/FTC/DRV/r (*n* = 860/4238; 20.3%) was the most frequent cART during the late period of 2011–2017. Most of the common first-line drug class combinations were NRTI/PI/boosted (*n* = 3682/8788; 41.9%), NRTI/NNRTI (*n* = 2951/8788; 33.6%), and NRTI/INSTI (*n* = 1676/8788; 19.1%), shown in Table 1. Since 2010, treatment initiation with NRTI/PI/boosted or NRTI/NNRTI decreased continuously and dropped below 5% in 2017. In comparison, treatment initiation with INSTI-based regimens increased constantly since 2008 and amounted to 85% of all patients initiating cART in 2017. Changes over time are shown in detail in Fig. 1.

### Durability of the first-line cART regimen

During 44,439 patient-years of follow-up and a median follow-up time of 3.83 years (IQR 1.30–7.81) per patient, the overall rate of first-line cART modification was 25 per 100 person-years. In total, 4210 (47.9%) patients modified their first-line therapy during follow-up. The median durability was 63 months (IQR 59–66), and was significantly longer in the early period compared to the late period (68 months, 95% CI 64–72 vs. 52 months, 95% CI 48–55; log-rank test *p* = 0.002) (Table S1).

The reason for modifying first-line therapy was recorded in 3597/4210 (85.4%) patients. The most commonly reported causes in men and women were side effects of drugs 792/3597 (22.0%), simplification of therapy 394/3597 (11.0%), patients’ choice 267/3597 (7.4%), decision of the responsible physician 259/3597 (7.2%), non-adherence 212/3597 (5.9%), comorbidities 136/3597 (3.8%), and virological failure 133/3597 (3.7%). Among women, side effects of drugs 152/588 (25.9%), patients’ choice 75/588 (12.8%), simplification of therapy 70/588 (11.9%), and pregnancy 51/588 (8.7%) were the most common causes to modify first-line therapy.

Physicians’ choice to modify first-line therapy was reported in 4.9% (128/1609) of patients during the early period and increased to 8.1% (131/2601) during the late period. The percentage of patients who modified first-line cART due to the simplification of therapy was lower in the early period than in the late period (192/2601; 7.4% vs. 202/1609; 12.6%).

Of the 4210 patients who modified their first-line cART, 21.5% (701/3259) switched from a MTR to a STR. The proportion increased from 19.4% (384/1981) in the early period to 24.8% (317/1278) during the late period (*p* < 0.001). A total of 31.3% (1084/3464) switched from a non-INSTI towards an INSTI-based regimen, the proportion increased from 24.6% (564/2297) to 44.6% (520/1167; *p* < 0.001) comparing the early to the late period.

### Factors associated with first-line cART durability

**Fig. 2:**
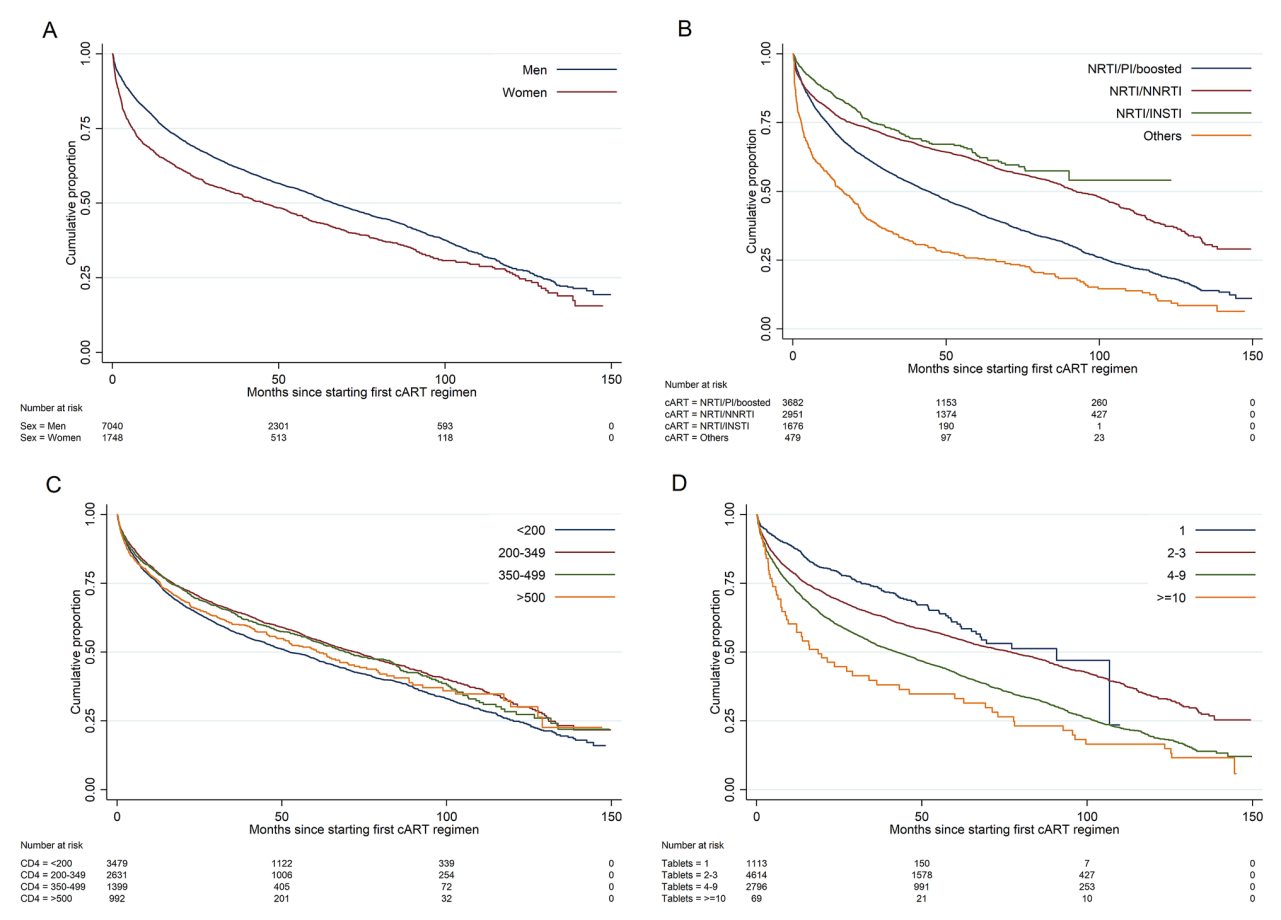
Unadjusted cumulative proportion of first-line cART durability. **a** Sex, **b** first-line drug class: NRTI; nucleoside reverse- transcriptase inhibitor, NNRTI; non-nucleoside reverse-transcriptase inhibitors INSTI; integrase inhibitor, PI; protease inhibitor. **c** Pre-cART CD4+ T-cell count and **d** number of tablets of the first-line cART regimen per day

Time-to-event analyses revealed prognostic factors to modify the first-line regimen, illustrated in the KM plots in Fig. 2a–d and Supplementary Figures S2 A and B and Table S1. For the duration of the first-line regimens, significant differences were identified among sex (*p* < 0.001), different drug classes (*p* < 0.001), and the year of cART initiation (*p* = 0.002). The median durability of the first-line regimen was significantly shorter in patients on MTR than in patients on STR (median 51 months, 95% CI 47–53 vs. 93 months, 95% CI 87–97; *p* < 0.001) (Table S1). KM analyses also revealed an increasing trend of first-line cART modification with a lower pre-cART CD4+ T-cell count (< 350 µL) (*p* < 0.001). These differences remained significant in the adjusted multivariable Cox regression model.

Women were more likely to modify the first-line regimen than men (aHR 1.24; 95% CI 1.12–1.37). Patients on a STR were significantly less likely to modify the first-line regimen compared to patients on an MTR (aHR 0.91; 95% CI 0.70–0.94). In addition, the frequency of tablet intake twice daily compared to once daily was significantly associated with treatment modification (aHR 1.34; 95% CI 1.22–1.48). We found that being on a NRTI/NNRTI (aHR 0.75; 95% CI 0.67–0.84) or a NRTI/INSTI (aHR 0.44; 95% CI 0.39–0.50) first-line regimen was associated with lower rates of modification, compared to being on a NRTI/PI/boosted first-line regimen. Modification of first-line regimen increased in the late period (2011–2017) compared to the early period (2005–2010) (aHR 1.45; 95% CI 1.33–1.58) (Table 2).

**Table 2:** Associations between baseline characteristics and first-line cART modification between 2005 and 2017

	Univariable model*		Mutivariable model*	
	HR (95% CI)	*p* value	aHR (95% CI)	*p* value
Age				
18–39				
40–69	1.02 (0.96–1.09)	0.519		
≥ 70	1.06 (0.77–1.48)	0.711		
Sex				
Female	1.32 (0.23–1.42)	**<** **0.001**	1.24 (1.12–1.37)	**<** **0.001**
Male				
Region of origin				
Germany				
Europe	1.12 (1.02–1.24)	**0.020**	1.06 (0.96–1.19)	0.256
Middle East	1.14 (0.85–1.52)	0.392	1.17 (0.86–1.95)	0.314
Sub-Saharan Africa	1.19 (1.09–1.31)	**<** **0.001**	0.94 (0.73–1.20)	0.621
Asia, Australia and New-Zealand	1.05 (0.88–1.25)	0.613	0.98 (0.79–1.23)	0.915
North and Latin America	0.89 (0.72–1.09)	0.521	0.90 (0.72–1.13)	0.371
Others/unknown	1.40 (1.11–1.69)	**0.003**	1.39 (1.09–1.77)	**0.007**
Transmission risk group				
MSM				
PWID	1.33 (1.16–1.52)	**<** **0.001**	1.04 (0.90–1.20)	0.597
HTS	1.09 (1.00–1.19)	**0.039**	0.98 (0.88–1.09)	0.726
ENDEMIC	1.26 (1.15–1.38)	**<** **0.001**	1.03 (0.81–1.31)	0.823
Other/unknown	1.18 (1.08–1.30)	**<** **0.001**	1.02 (0.92–1.13)	0.751
Pre-cART CD4+ T-cell count (µL)				
< 200				
200–349	0.82 (0.76–0.88)	**<** **0.001**	0.92 (0.85–0.99)	**0.037**
350–499	0.85 (0.77–0.93)	**<** **0.001**	0.97 (0.88–1.07)	0.557
≥ 500	0.94 (0.84–1.05)	0.242	1.07 (0.95–1.21)	0.250
Pre-cART HIV-1 RNA viral load (copies/mL)				
< 200	0.97 (0.79–1.20)	0.789	0.90 (0.71–1.14)	0.377
201–5000	0.95 (0.86–1.06)	0.373	0.82 (0.66–1.02)	0.079
5001–100,000	0.85 (0.79–0.91)	**<** **0.001**	0.93 (0.75–1.16)	0.509
> 100,000				
First-line drug class				
NRTI/PI/boosted				
NRTI/NNRTI	0.61 (0.57–0.65)	**<** **0.001**	0.75 (0.67–0.84)	**<** **0.001**
NRTI/INSTI	0.52 (0.46–0.57)	**<** **0.001**	0.44 (0.39–0.50)	**<** **0.001**
Others	1.67 (1.50–1.87)	**<** **0.001**	1.41 (1.23–1.62)	**<** **0.001**
Tablet regimen				
STR	0.64 (0.59–0.68)	**<** **0.001**	0.91 (0.70–0.94)	**0.041**
MTR				
Year of cART initiation				
2005–2010				
2011–2017	1.11 (1.04–1.19)	**0.002**	1.45 (1.33–1.58)	**<** **0.001**
INSTI regimen**				
DTG				
RAL	2.01 (1.59–2.53)	**<** **0.001**		
EVG	1.46 (1.08–1.96)	**0.013**		
Tablet intake				
Once per day				
Twice per day	1.42 (1.34–1.52)	**<** **0.001**	1.34 (1.22–1.48)	**<** **0.001**

*p* values < 0.05 in bold depict significant results

Risk group: *MSM* men who have sex with men, *HTS* heterosexual, *ENDEMIC* recent immigration from a country with a high HIV prevalence > 1%, *PWID* people who inject drugs. First-line drug class: *NRTI* nucleoside reverse-transcriptase inhibitor, *NNRTI* non-nucleoside reverse-transcriptase inhibitors, *INSTI* integrase strand transfer inhibitors, *PI* protease inhibitor. Substance of first-line regimen: *TDF* tenofovir, *FTC* emtricitabine, *EFV* efavirenz, *DRV* darunavir, *ATV* atazanavir, *RAL* raltegravir, *NVP* nevirapine, *RPV* rilpivirine, *DTG* dolutegravir. Tablet regimen: *STR* single-tablet regimen, *MTR* multi-tablet regimen. First-line with INSTI regimen: *RAL* raltegravir, *EVG* elvitegravir, *DLG* dolutegravir

*Results from a Cox proportional hazards model displayed with adjusted Hazard ratios (aHRs) and 95% confidence intervals (CI)

**Variable excluded from multivariable analysis due to multicollinearity

We also identified significant differences for modification of the discontinuation of first-line cART among transmission risk groups and different INSTI regimens. Patients on RAL and EVG were significantly more likely to modify therapy compared to those on DTG (HR 2.01; 95% CI 1.59–2.53 and HR 1.46; 95% CI 1.08–1.96, respectively). However, the transmission risk group did not remain significant in the adjusted multivariable Cox regression model, and INSTI regimens were excluded due to multicollinearity. Uni-and multivariable analyses were also performed separately for the early and late period. Details are displayed in supplementary table S2–S4.

### Antiviral efficacy and immunological recovery after first-line initiation

Among patients with an available HIV-1 RNA assessment at month 12 (± 6 months), 5745/6089 (95.4%) achieved viral suppression (< 200 copies/mL).

Pre-cART viral load was significantly higher in patients who modified their first-line cART regimen within 12 months (± 6 months) (median 70,324 copies/mL; IQR 12,812–257,914 copies/mL) than in patients who remained on their first-line regimen (median 63,126 copies/mL; IQR 14,078–200,000; *p* = 0.013). In the whole group, the median decrease in viral load after 12 months on cART was 3.4 log copies/mL (IQR 2.8–4.0 log copies/mL) and was greater in those who remained on their first-line cART than in patients who modified the initial treatment (4.0 vs. 3.5 log copies/mL, respectively). The overall pre-cART CD4+ T-cell count increased with a median gain of 205 cells/µL (IQR 180–374 cells/µL) at month 12 (± 6 months), a relative increase of 84%. No differences were observed between individuals who remained on their first-line regimen compared to those who modified their first-line regimen (*p* = 0.843).

## Discussion

This study examined key factors for durability of first-line cART treatment in routine clinical care conditions across Germany. We found several factors that were associated with a shorter durability of first-line cART in the multivariable analysis including being female, low CD4+ T-cell counts at the beginning of treatment, PI-based cART, MTR, cART initiation in the late period (2011–2017), and tablet intake more than once a day.

The significant difference in sex, as well as in people presenting with low pre-cART CD4+ T-cell count, may be related to non-adherence that is more frequently seen in female PLWH and individuals originating from sub-Saharan Africa, as well as in late presenters [19]. In our study population, most people from sub-Saharan Africa were women (70%). In addition, pregnancies, which accounted for about 10% of reported reasons for treatment modification, might also contribute to the lower durability of cART that is seen in women [20].

Interestingly, in our study, there was no tendency towards a better durability of first-line therapy in recent years as it was found by other studies [7, 8]. The most common causes of modification in our cohort were side effects of drugs, simplification of therapy, and patient’s choice. Thus, we hypothesize that the newer INSTI-class and additional STR options in the late period offered an interesting alternative for patients and physicians, contributing to higher rates of modification [21]. This is confirmed by our observation that physicians’ choice as well as simplification of therapy as reasons for treatment switch were significantly more frequent in the late period between 2011 and 2017.

Most subjects, especially in the early period, received PI-based cART regimens, which showed a higher risk for first-line modification compared to NNRTI- or INSTI-based regimens. These results are in line with previous studies, showing an inferiority of PI-based regimens compared to NNRTI- or INSTI-based regimens in terms of treatment [22, 23]. This might be due to more interactions with other medications and higher rates of side effects. However, higher costs and strategic changes in therapy could also partially explain higher rates of modification of PI-based regimens. In addition, PI-based STR has only been available since 2017, so they may be underrepresented in our cohort.

Once-daily regimens, especially those with an STR, showed a better durability of the first-line treatment, as also shown in other studies [4, 10, 24, 25]. This effect is most likely due to the potentially improved adherence that is seen with these treatment regimens [26] and also due to the lack of further optimization options.

From 2014 onwards, INSTI-based regimens already accounted for the majority of initial treatments resulting in a maximum of 86% in the year 2017. We found a higher risk for modification on RAL compared to EVG and DTG, mainly due to therapy simplification, which is comparable to previous studies [27, 28]. Furthermore, we found that patients who received INSTI-based regimens as their first-line treatment had the highest probability of an undetectable viremia after 12 months. Thus, these data underline the current role of INSTI-based regimens as recommended first-line treatments [1]. However, even in our study, long-term data of INSTI-based regimens are still rare.

Our study had a couple of limitations. First, these are retrospective, “real-life” data predominantly clinical HIV centers, which occupy a key position in outpatient care in the German health care system. In our analysis, patients had a median age of 38 years and were predominantly men (80.1%), of which about one third (63.5%) reported to be MSM. These baseline characteristics did not change notably over time comparing the early period of 2005–2010 with the late period of 2011–2017. Thus, while our study was limited to PLWH in Germany, the characteristics of patients starting their first-line antiretroviral treatment compare very well with those reported in other cohorts in the USA, France, and Australia and, therefore, seem to be representative for PLWH seen in outpatient clinics in industrialized countries [7–9]. Second, we were lacking data about severe medical preconditions such as prior AIDS-defining diseases of these patients that could have favored some cART regimens.

In conclusion, overall efficacy of first-line treatment was good, with 95.4% achieving viral suppression 1 year after starting cART. Amongst the different classes, INSTI-based regimens showed superiority in terms of durability. Thus, our data confirm the rationale of the current guidelines that recommend INSTI-based regimens as first-line treatment in HIV-infected patients. However, future studies are required to assess the efficacy and durability of these treatments over a longer period. Recent studies observed high modification rates of INSTIs, in particular for DTG, due to adverse events and weight gain [29, 30].

We elucidated several factors that were significantly associated with the modification of first-line regimens. The modification rates were higher during the late period, particularly among women, and in patients on an MTR. Patients at higher risk for treatment modification might require more frequent follow-up visits and better monitoring during their first-line cART regimen.

## Electronic supplementary material

Below is the link to the electronic supplementary material.

Supplementary material 1 (DOCX 179 kb)

Supplementary material 2 (DOCX 36 kb)
