# Correction to: Treatment modifcation after starting cART in people living with HIV: retrospective analysis of the German ClinSurv HIV Cohort 2005–2017

**DOI:** 10.1007/s15010-021-01655-0

**Published:** 2021-08-03

**Authors:** Melanie Stecher, Philipp Schommers, Christian Kollan, Matthias Stoll, Frieder Kuhlendahl, Hans-Jürgen Stellbrink, Jan-Christian Wasmuth, Christoph Stephan, Laura Hamacher, Clara Lehmann, Christoph Boesecke, Johannes Bogner, Stefan Esser, Carlos Fritzsche, Annette Haberl, Dirk Schürmann, Olaf Degen, Heinz-August Horst, Christian Hoffmann, Björn Jensen, Carolynne Schwarze-Zander, Martin Platten, Gerd Fätkenheuer, Daniel Schmidt, Barbara Gunsenheimer-Bartmeyer, Jörg Janne Vehreschild, On behalf of the ClinSurv Study Group

**Affiliations:** 1 Department I for Internal Medicine, Faculty of Medicine and University Hospital Cologne, University of Cologne, Herderstraße 52-54, 50931 Cologne, Germany; 2 German Center for Infection Research (DZIF), Partner Site Bonn-Cologne, Cologne, Germany; 3 Robert-Koch Institute (RKI), Berlin, Germany; 4 Department of Clinical Immunology and Rheumatology, Hannover Medical School, Hannover, Germany; 5 Ifi-Institute for Interdisciplinary Medicine, Hamburg, Germany; 6 Infectious Disease Medical Center, Hamburg, Germany; 7 Department I for Internal Medicine, University Hospital of Bonn, Bonn, Germany; 8 German Center for Infection Research (DZIF), Partner Site Bonn-Cologne, Bonn, Germany; 9 Department of Infectious Diseases, University Hospital Frankfurt, Goethe-University, Frankfurt am Main, Germany; 10 Section for Infectious Diseases, Medical Clinic and Polyclinic IV, University Hospital, Ludwig-Maximilians-University Munich, Munich, Germany; 11 Clinic of Dermatology, Department of Venerology, University Hospital Essen, Essen, Germany; 12 Department of Tropical Medicine and Infectious Diseases, University of Rostock, Rostock, Germany; 13 ICH Study Center, Infektionsmedizinisches Centrum Hamburg, Hamburg, Germany; 14 Department of Gastroenterology, Hepatology and Infectiology, University of Düsseldorf, Düsseldorf, Germany; 15 Labor Dr. Wisplinghoff, Cologne, Germany; 16 Charité - University Medicine Berlin, Berlin, Germany; 17 Department of Hematology and Oncology, Johann Wolfgang Goethe University of Frankfurt, Frankfurt am Main, Germany; 18 University Clinic Hamburg Eppendorf, Hamburg, Germany; 19 University Hospital Schleswig–Holstein, Kiel, Germany

## Correction to: Infection (2020) 48:723–733 10.1007/s15010-020-01469-6

The original version of this article unfortunately contained a mistake.

In this article the authors Dirk Schürmann at affiliation Charité, University Medicine, Berlin, Olaf Degen at affiliation University Clinic Hamburg Eppendorf, Hamburg and Heinz-August Horst at affiliation University Hospital Schleswig–Holstein, Kiel, Germany were missing from the author list.

The original article has been corrected.

