# Supplementary material for: Treatment modification after starting cART in people living with HIV: retrospective analysis of the German ClinSurv HIV Cohort 2005–2017
Source: Infection. 2020 Jul 1;48(5):723–33. doi: 10.1007/s15010-020-01469-6 (PMC7519003; doi:10.1007/s15010-020-01469-6)
Supplement: Supplementary file 1 — Supplementary material 1 (DOCX 179 kb) [file 15010_2020_1469_MOESM1_ESM.docx]

## Supplements


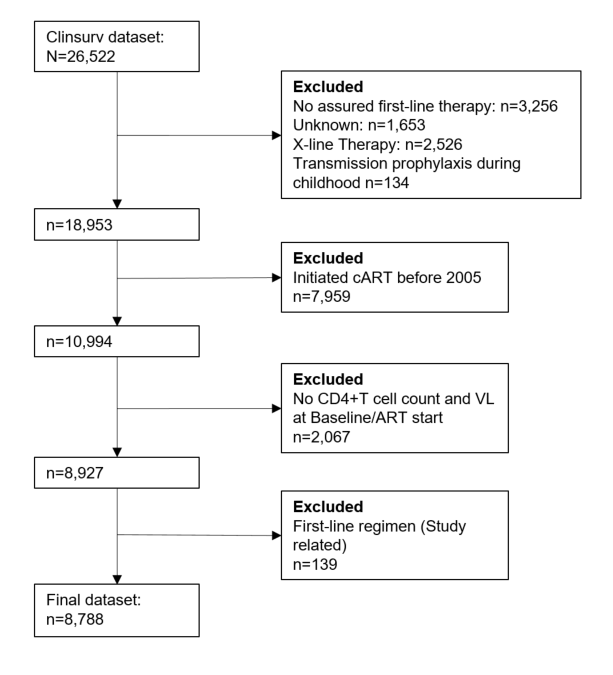
Figure S1. Flow chart of selected cases due to predefined in- and exclusion criteria.

*
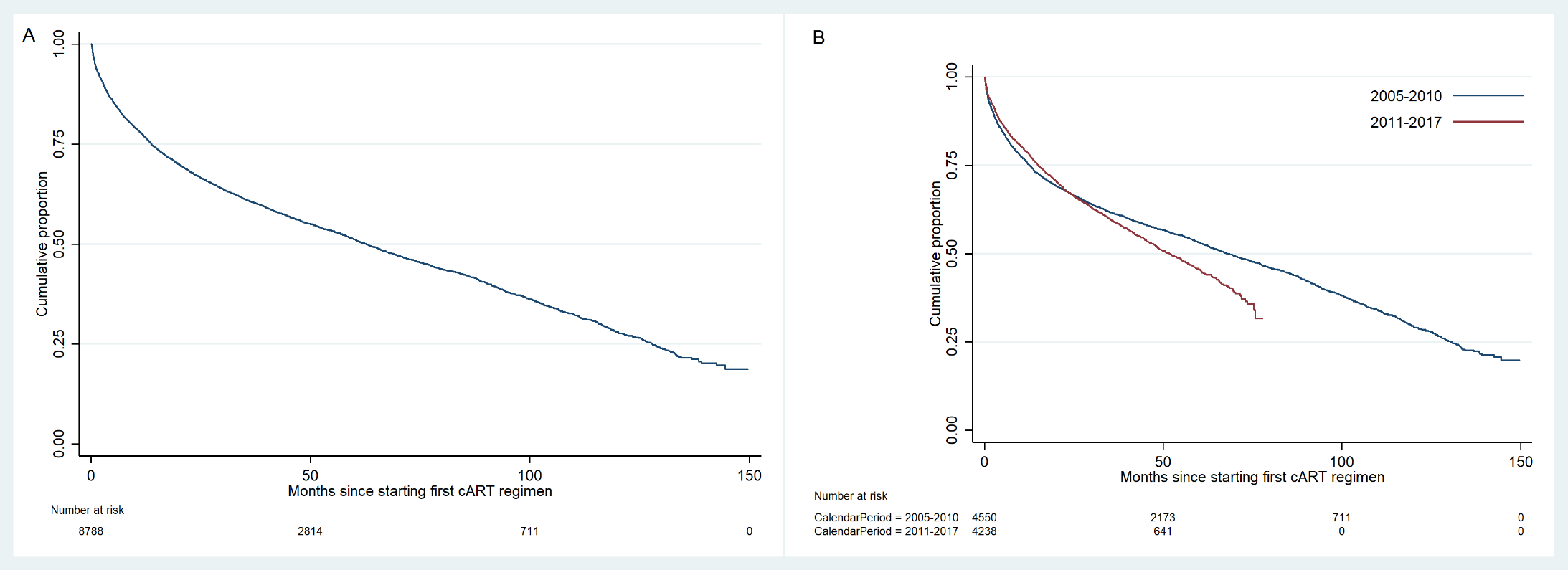
*

Figure S2 A&B. Unadjusted cumulative proportion of first-line cART durability A) Overall and stratified by B) Year of first-line initiation, calendar period 2005-2010 and 2011-2017.
