# Supplementary material for: Treatment modification after starting cART in people living with HIV: retrospective analysis of the German ClinSurv HIV Cohort 2005–2017
Source: Infection. 2020 Jul 1;48(5):723–33. doi: 10.1007/s15010-020-01469-6 (PMC7519003; doi:10.1007/s15010-020-01469-6)
Supplement: Supplementary file 2 — Supplementary material 2 (DOCX 36 kb) [file 15010_2020_1469_MOESM2_ESM.docx]

Table S1. Durability of the first-line cART regimen (months) among different subgroups between 2005 and 2017.

|  |  |  |
| --- | --- | --- |
|  |  |  |
|  | Durability of first-line cART (median time in months, IQR) | p-value* |
|  |  |  |
| **Total study population** | 63 (59-66) |  |
| **Age** |  | 0.772 |
| 18-39 | 63 (59-68) |  |
| 40-69 | 60 (56-65) |  |
| ≥70 | 49 (26-72) |  |
| **Sex** |  | **<0.001** |
| Female | 45 (38-51) |  |
| Male | 66 (62-69) |  |
| **Region of origin** |  | **<0.001** |
| Germany | 66 (62-69) |  |
| Europe | 58 (50-66) |  |
| Middle East | 58 (32-84) |  |
| Sub-Saharan Africa | 53 (42-64) |  |
| Asia, Australia & New-Zealand | 60 (40-80) |  |
| North and Latin America | 64 (20-108) |  |
| Others/Unknown | 39 (18-62) |  |
| **Transmission risk group** |  | **<0.001** |
| MSM | 67 (62-72) |  |
| HTS | 62 (55-69) |  |
| ENDEMIC | 53 (44-62) |  |
| PWID | 47 (34-61) |  |
| Other/ Unknown | 56 (47-65) |  |
| **Pre-cART CD4+ T-cell count (µL)** |  | **<0.001** |
| <200 | 52 (47-57) |  |
| 200 – 349 | 72 (66-78) |  |
| 350 – 499 | 69 (60-78) |  |
| ≥500 | 61 (51-71) |  |
| **Pre-cART HIV-1 RNA viral load (copies/ml)** |  | **<0.001** |
| 51-200 | 63 (53-74) |  |
| 201-5,000 | 63 (52-73) |  |
| 5,001-100,000 | 70 (65-75) |  |
| 100,001 - 1 Mio. | 58 (53-64) |  |
| >1Mio. | 30 (24-36) |  |
| **First-line drug class** |  | **<0.001** |
| NRTI/PI/boosted | 59 (57-61) |  |
| NRTI/NNRTI | 82 (77-86) |  |
| NRTI/INSTI | 83 (85-92) |  |
| Others | 19 (15-23) |  |
| **Number of tablets per day** |  | **<0.001** |
| 1 | 90 (77-103) |  |
| 2-3 | 76 (71-82) |  |
| 4-9 | 42 (38-46) |  |
| ≥10 | 18 (7-29) |  |
| **Tablet regimen** |  | **<0.001** |
| STR | 93 (87-97) |  |
| MTR | 51 (47-53) |  |
| **Year of cART initiation** |  | **0.002** |
| 2005-2010 | 68 (63-72) |  |
| 2011-2017 | 51 (48-55) |  |
| **Tablet intake** |  | **<0.001** |
| Once per day | 71 (66-75) |  |
| Twice per day | 40 (35-46) |  |
|  |  |  |

*Risk group: MSM, men who have sex with men; HTS, heterosexual; ENDEMIC, recent immigration from a country with a high HIV prevalence; PWID, people who inject drugs. First-line drug class: NRTI; nucleoside reverse- transcriptase inhibitor, NNRTI; non-nucleoside reverse-transcriptase inhibitors INSTI; integrase strand transfer inhibitors, PI; protease inhibitor. Tablet regimen: STR, single tablet regimen and MTR multi tablet regimen.*

**Durability of first-line regimen are displayed with median and interquartile range (IQR); Log-rank overall comparison was used to compare different subgroups (p<0.05).*

*n.a.: the median duration has not been reached.*

Table S2. Durability of the first-line cART regimen (months) and tablet regimens during the time between 2011 and 2017.

|  |  |  |
| --- | --- | --- |
|  |  |  |
|  | Durability of first-line cART (median time in months, IQR) | p-value* |
|  |  |  |
| **First-line drug class** |  | **<0.001** |
| NRTI/PI/boosted | 47 (34-61) |  |
| NRTI/NNRTI | 79 (61-97) |  |
| NRTI/INSTI | 90 (52-128) |  |
| Others | 12 (7-26) |  |
| **Tablet regimen** |  | **<0.001** |
| STR | 88 (71-105) |  |
| MTR | 48 (37-59) |  |
|  |  |  |

*NRTI; nucleoside reverse- transcriptase inhibitor, NNRTI; non-nucleoside reverse-transcriptase inhibitors INSTI; integrase strand transfer inhibitors, PI; protease inhibitor. Tablet regimen: STR, single tablet regimen and MTR multi tablet regimen.*

Supplemental Table S3. Associations between baseline characteristics and first-line cART modification between the early period (2005 and 2010).

|  |  | |  | |  |  |
| --- | --- | --- | --- | --- | --- | --- |
|  | Univariable model* | | | | Mutivariable model* | |
|  |  | HR (95% CI) | | p-value | aHR (95% CI) | p-value |
| **Age** |  | |  | |  |  |
| 18-39 |  | |  | |  |  |
| 40-69 | 0.98 (0.91-1.06) | | 0.675 | |  |  |
| ≥70 | 1.08 (0.67-1.74) | | 0.752 | |  |  |
| **Sex** |  | |  | |  |  |
| Female | 1.23 (1.12-1.35) | | **<0.001** | | 1.58 (0.64-3.91) | 0.323 |
| Male |  | |  | |  |  |
| **Region of origin** |  | |  | |  |  |
| Germany |  | |  | |  |  |
| Europe | 1.04 (0.91-1.18) | | 0.554 | | 0.89 (0.34-2.34) | 0.809 |
| Middle East | 1.01 (0.67-1.52) | | 0.975 | | n.a. |  |
| Sub-Saharan Africa | 1.06 (0.94-1.19) | | 0.372 | | 0.16 (0.02-1.40) | 0.099 |
| Asia, Australia & New-Zealand | 1.12 (0.91-1.37) | | 0.301 | | 0.57 (0.07-4.82) | 0.608 |
| North and Latin America | 0.48 (0.57-0.98) | | **0.035** | | 9.16 (1.92-43.63) | 0.005 |
| Others/Unknown | 1.33 (0.94-1.87) | | 0.106 | | 0.56 (0.10-2.90) | 0.482 |
| **Transmission risk group** |  | |  | |  |  |
| MSM |  | |  | |  |  |
| PWID | 1.39 (1.19-1.63) | | **<0.001** | | 9.70 (1.41-6.67) | **0.021** |
| HTS | 1.09 (0.94-1.21) | | 0.138 | | 1.62 (0.67-3.92) | 0.288 |
| ENDEMIC | 1.13 (1.01-1.27) | | **0.033** | | 5.08 (0.79-32.87) | 0.088 |
| Other/ Unknown | 1.08 (0.96-1.22) | | 0.204 | | 0.87 (0.34-2.26) | 0.781 |
| **Pre-cART CD4+ T-cell count (µL)** |  | |  | |  |  |
| <200 |  | |  | |  |  |
| 200 – 349 | 0.87 (0.79-0.95) | | **0.003** | | 0.64 (0.28-1.48) | **0.299** |
| 350 – 499 | 0.97 (0.86-1.09) | | 0.589 | | 1.08 (0.53-2.23) | 0.829 |
| ≥500 | 1.14 (0.97-1.34) | | 0.107 | | 0.59 (0.14-2.50) | 0.476 |
| **Pre-cART HIV-1 RNA viral load (copies/ml)** |  | |  | |  |  |
| <200 | 0.89 (0.67-1.17) | | 0.405 | |  |  |
| 201-5,000 | 0.85 (0.66-1-09) | | 0.201 | |  |  |
| 5,001-100,000 | 0.89 (0.69-1.15) | | 0.365 | |  |  |
| >100,000 |  | |  | |  |  |
| **First-line drug class** |  | |  | |  |  |
| NRTI/PI/boosted |  | |  | |  |  |
| NRTI/NNRTI | 0.46 (0.59-0.69) | | **<0.001** | |  |  |
| NRTI/INSTI | 0.40 (0.29-0.54) | | **<0.001** | |  |  |
| Others | 1.74 (1.53-1.99) | | **<0.001** | | 6.39 (3.12-12.74) | **<0.001** |
| **Tablet regimen** |  | |  | |  |  |
| STR | 0.66 (0.61-0.73) | | **<0.001** | |  |  |
| MTR |  | |  | |  |  |
| **Year of cART initiation** | 0.98 (0.96-1.01) | | 0.196 | | 0.93 (0.64-1.35) | 0.697 |
| **INSTI regimen** |  | |  | |  |  |
| DTG |  | |  | |  |  |
| RAL | 3.00 (0.93-9.62) | | 0.065 | | 2.43 (0.71-8.25) | 0.156 |
| EVG | 8.27 (0.85-80.23) | | **0.069** | | 2.16 (0.18-25.95) | 0.543 |
| **Tablet intake** |  | |  | |  |  |
| Once per day |  | |  | |  |  |
| Twice per day | 1.56 (1.45-1.69) | | **<0.001** | | n.a. |  |
|  |  | |  | |  |  |

*Risk group: MSM, men who have sex with men; HTS, heterosexual; ENDEMIC, recent immigration from a country with a high HIV prevalence; PWID, people who inject drugs. First-line drug class: NRTI; nucleoside reverse- transcriptase inhibitor, NNRTI; non-nucleoside reverse-transcriptase inhibitors INSTI; integrase strand transfer inhibitors, PI; protease inhibitor. Substance of first-line regimen: TDF, Tenofovir; FTC, Emricitabine; EFV, Efavirenz; DRV, Darunavir; ATV, Atzanavir; RAL, Raltegravir; NVP, Nevirapin; RPV, Rilpirivin; DTG, Dolutegravir. Tablet regimen. STR, single tablet regimen and MTR multi tablet regimen. First-line with INSTI regimen: RAL, Raltegravir; EVG, Elvitegravir; DLG, Dolutegravir.*

**Results from a Cox proportional hazards model displayed with adjusted Hazard ratios (aHRs) and 95% confidence intervals (CI); n.a.: not applicable*

***Variable excluded from multivariable analysis due to multicollinearity.*

Supplemental Table S4. Associations between baseline characteristics and first-line cART modification between the later period (2011 and 2017).

|  |  | |  | |  |  |
| --- | --- | --- | --- | --- | --- | --- |
|  | Univariable model* | | | | Mutivariable model* | |
|  |  | HR (95% CI) | | p-value | aHR (95% CI) | p-value |
| **Age** |  | |  | |  |  |
| 18-39 |  | |  | |  |  |
| 40-69 | 1.09 (0.98-1.20) | | 0.111 | | 1.18 (0.92-1.51) | 0.182 |
| ≥70 | 1.02 (0.65-1.61) | | 0.919 | | 0.54 (0.13-2.21) | 0.393 |
| **Sex** |  | |  | |  |  |
| Female | 1.51 (0.35-1.69) | | **<0.001** | | 1.38 (0.97-1.98) | 0.077 |
| Male |  | |  | |  |  |
| **Region of origin** |  | |  | |  |  |
| Germany |  | |  | |  |  |
| Europe | 1.26 (1.09-1.46) | | **0.002** | | 1.22 (0.86-1.72) | 0.267 |
| Middle East | 1.32 (0.87-1.99) | | 0.919 | | 2.16 (0.98-4.77) | 0.056 |
| Sub-Saharan Africa | 1.48 (1.28-1.72) | | **<0.001** | | 1.46 (0.47-4.48) | 0.510 |
| Asia, Australia & New-Zealand | 0.89 (0.64-1.25) | | 0.499 | | 1.31 (0.53-3.25) | 0.556 |
| North and Latin America | 1.19 (0.87-1.63) | | 0.272 | | 1.88 (1.04-3.41) | **0.038** |
| Others/Unknown | 1.42 (1.09-1.85) | | **0.009** | | 1.59 (0.86-2.98) | 0.138 |
| **Transmission risk group** |  | |  | |  |  |
| MSM |  | |  | |  |  |
| PWID | 1.16 (0.91-1.49) | | 0.234 | | 1.54 (0.89-2.66) | 0.127 |
| HTS | 1.12 (0.98-1.29) | | 0.099 | | 0.80 (0.56-1.16) | 0.246 |
| ENDEMIC | 1.57 (1.36-1.81) | | **<0.001** | | 1.00 (0.34-2.95) | 0.997 |
| Other/ Unknown | 1.37 (1.19-1.58) | | **<0.001** | | 0.99 (0.70-1.38) | 0.930 |
| **Pre-cART CD4+ T-cell count (µL)** |  | |  | |  |  |
| <200 |  | |  | |  |  |
| 200 – 349 | 0.69 (0.62-0.79) | | **<0.001** | | 0.79 (0.59-1.08) | **0.022** |
| 350 – 499 | 0.66 (0.57-0.76) | | **<0.001** | | 0.83 (0.59-1.15) | 0.408 |
| ≥500 | 0.73 (0.62-0.84) | | **<0.001** | | 0.89 (0.71-1.38) | 0.966 |
| **Pre-cART HIV-1 RNA viral load (copies/ml)** |  | |  | |  |  |
| <200 | 1.17 (0.79-1.34) | | 0.435 | | 1.12 (0.52-2.41) | 0.768 |
| 201-5,000 | 0.94 (0.65-1.36) | | 0.732 | | 1.15 (0.58-2.29) | 0.689 |
| 5,001-100,000 | 1.33 (0.91-1.92) | | 0.136 | | 1.44 (0.72-2.86) | 0.303 |
| >100,000 |  | |  | |  |  |
| **First-line drug class** |  | |  | |  |  |
| NRTI/PI/boosted |  | |  | |  |  |
| NRTI/NNRTI | 0.56 (0.49-0.63) | | **<0.001** | | 0.79 (0.64-0.99) | 0.041 |
| NRTI/INSTI | 0.48 (0.42-0.55) | | **<0.001** | | 0.49 (0.42-0.57) | **<0.001** |
| Others | 1.59 (1.31-1.96) | | **<0.001** | | 1.99 (1.32-3.01) | **0.001** |
| **Tablet regimen** |  | |  | |  |  |
| STR | 0.59 (0.52-0.66) | | **<0.001** | | 0.16 (0.59-0.42) | **<0.001** |
| MTR |  | |  | |  |  |
| **Year of cART initiation** | 1.01 (0.97-1.04) | | 0.788 | | 1.11 (1.01-1.23) | **0.024** |
| **INSTI regimen** |  | |  | |  |  |
| DTG |  | |  | |  |  |
| RAL | 2.11 (1.66-2.69) | | **<0.001** | | 8.37 (5.96-1.18) | **<0.001** |
| EVG | 1.39 (1.04-1.88) | | **0.028** | | 10.11 (3.84-26.61) | **<0.001** |
| **Tablet intake** |  | |  | |  |  |
| Once per day |  | |  | |  |  |
| Twice per day | 1.31 (1.16-1.49) | | **<0.001** | | n.a. |  |
|  |  | |  | |  |  |

*Risk group: MSM, men who have sex with men; HTS, heterosexual; ENDEMIC, recent immigration from a country with a high HIV prevalence; PWID, people who inject drugs. First-line drug class: NRTI; nucleoside reverse- transcriptase inhibitor, NNRTI; non-nucleoside reverse-transcriptase inhibitors INSTI; integrase strand transfer inhibitors, PI; protease inhibitor. Substance of first-line regimen: TDF, Tenofovir; FTC, Emricitabine; EFV, Efavirenz; DRV, Darunavir; ATV, Atzanavir; RAL, Raltegravir; NVP, Nevirapin; RPV, Rilpirivin; DTG, Dolutegravir. Tablet regimen. STR, single tablet regimen and MTR multi tablet regimen. First-line with INSTI regimen: RAL, Raltegravir; EVG, Elvitegravir; DLG, Dolutegravir*

**Results from a Cox proportional hazards model displayed with adjusted Hazard ratios (aHRs) and 95% confidence intervals (CI).*

***Variable excluded from multivariable analysis due to multicollinearity.*
